# The prognostic importance of the global immune-nutrition-information index (GINI) in patients with Ras wild type metastatic colorectal cancer

**DOI:** 10.1038/s41598-025-11148-x

**Published:** 2025-07-15

**Authors:** Oktay Bozkurt, Rıdvan Gönül, Bugra Umut Kaya, Gozde Erturk Zararsiz, Mevlüde İnanc, Metin Özkan

**Affiliations:** 1 Department of Medical Oncology, Erciyes University Faculty of Medicine, Kayseri, Turkey; 2 Department of Internal Medicine, Erciyes University Faculty of Medicine, Kayseri, Turkey; 3 Department of Biostatistics, Erciyes University School of Medicine, Kayseri, Türkiye Turkey

**Keywords:** GINI, Colorectal cancer, Prognosis, Cancer, Oncology

## Abstract

In this study, we aimed to evaluate the clinical impact of the Global Immune-Nutrition Information Index (GINI) in patients with Ras wild-type metastatic colorectal cancer (mCRC) who received first-line palliative chemotherapy Ras wild-type mCRC. We retrospectively reviewed 177 patients diagnosed with Ras Wild Type mCRC between March 2009 and December 2023. The GINI was defined as follows: GINI= [C-reactive protein×platelet×monocyte×neutrophil]/[albumin×lymphocyte]. According to threshold values determined by receiver operating characteristics (ROC) analysis, the GINI was divided into two groups with < 2000 and ≥ 2000. Survival probabilities were predicted with the Kaplan-Meier method and group comparisons were applied with the Log-rank test. Furthermore, univariate and multiple Cox regression analyses were used to determine the most substantial risk elements. Median progression-free survival (PFS) 15 months in the group with GINI ≥ 2000 (95% Confidence interval (CI): 10.5–19.48) and 27 months (95% CI: 17.5–36.4) in the group with GINI < 2000 (*p* = 0.00021). The median OS was 27 months (95% CI: 23.8–30.1) in the GINI ≥ 2000 group and 77 months (95% CI: 59.7–94.2) in the GINI < 2000 group (*p* < 0.001). The results of multivariate analysis for PFS showed that albumin (HR, 1.83, *p* = 0.009), and prechemotherapy GINI (HR 1.79, *p* = 0.004) were significant independent prognostic factors. The results of multivariate analysis for OS showed that performance status (HR, 1.65; *p* = 0.018), number of metastatic sites (HR, 1.74, 0.005), skin toxicity (HR, 1.79, *p* = 0.003), pre-chemotherapy NLR (HR, 1.49, *p* = 0.045) and prechemotherapy GINI (HR 3.25, *p* < 0.001) were significant independent prognostic factors. Higher GINI values were associated with worse survival outcomes in patients with RAS wild-type mCRC, supporting its potential clinical use as a prognostic biomarker.

## Introduction

Colorectal cancer is recognized as the fourth most prevalent malignant neoplasm and the third primary driver of cancer mortality across the globe^1^. Approximately 20% of colorectal cancers (CRCs) are in the metastatic stage at diagnosis, and approximately half of those with localized disease progress to the metastatic stage at diagnosis^2^. Overall survival (OS) in metastatic colorectal cancer (mCRC) has been enhanced by new treatment strategies developed through a better understanding of the biology of the disease and improvements in supportive care^3^.

Numerous studies have shown that RAS mutations occur in approximately 50% of patients with CRC and play a crucial role in the progression of CRC. Recently, some research has indicated that mutated RAS may have a negative impact on prognosis compared to wild-type RAS in mCRC patients undergoing standard first-line chemotherapy treatments^4,5^.

Additionally, recent findings have indicated that certain KRAS mutations are linked to chemoresistance in pancreatic cancer. Specifically, pG12D mutants exhibited greater resistance to chemotherapy than wtKRAS, p.G12C, and p.G12V mutants^6^.

These findings indicate that oncogenic KRAS mutations exhibit distinct functional characteristics and may play a role in diverse biological and clinical manifestations of cancers. Therefore, we included patients with RAS wild-type colorectal cancer to create a more homogeneous group.

In metastatic colorectal cancer (mCRC), the Ras mutational status is a critical factor in determining patient eligibility for anti-EGFR monoclonal antibody therapies like cetuximab and panitumumab^7^. Previous study has demonstrated that chemotherapy combined with targeted agents has a survival advantage over chemotherapy alone in mCRC^8^.

Neutrophils are considered to be effective in tumor cell proliferation, vascularization, and metastasis through the production of proangiogenic chemokines and cytokines^9^. The prognosis of cancer patients is significantly affected by lymphocytes, which work by preventing tumor cell growth, movement, and invasion^10,11^. In addition to hemostasis and thrombosis, platelets play a role in cancer cell proliferation and metastasis through the secretion of numerous proangiogenic molecules and proteases^12^.

The patient’s nutritional status affects albumin levels, a negative acute-phase reactant synthesized by the liver. Albumin level is a prognostic variable for survival in colorectal cancer^13^. It is an acute-phase protein that is an indicator of tissue damage and its synthesis is affected by many factors, including interleukin-1 and tumor necrosis factor^14^. Several previous investigations have reported the importance of CRP as a prognostic tool for CRC^15,16^.

Recent research shows that certain inflammation and nutrition markers, like NLR, platelet-to-lymphocyte ratio, PNI, and CONUT score, can influence how diseases progress^17–20^.

Topkan et al. first demonstrated that the GINI index, which can simultaneously assess nutritional and inflammatory status, is a beneficial prognostic tool for patients with stage IIIc NSCLC treated with chemoradiotherapy^21^.

Drawing from these results, we proposed that GINI could be beneficial in managing mCRC. Since chemotherapy is administered to mCRC patients, it is believed that inflammation and nutritional status before treatment are crucial. Nonetheless, there have been no studies assessing the prognostic significance of GINI in a particular group of RAS wild-type mCRC patients.

This study aimed to determine the clinical importance of GINI in patients with RAS wild-type mCRC treated with first-line palliative chemotherapy and to investigate the role of GINI as a potential outcome predictor in the treatment of mCRC.

## Materials and methods

### Patient data

Between March 2009 and September 2023, 177 patients with RAS wild-type mCRC treated with first-line palliative therapy were included in this single-center, retrospective study. This study received ethical approval, including a waiver of informed consent due to its retrospective nature, from the Erciyes University Health Sciences Research Ethics Committee, Melikgazi/Kayseri, Turkey (Decision Number: 2025/127).

GINI was determined using the following formula: GINI=(C-reactive protein × platelets × monocytes × neutrophils)/(albumin × lymphocytes)^21^.

Blood tests, such as complete blood count and blood chemistry, were conducted 2–24 h prior to chemotherapy. Complete blood count was assessed using the Sysmex XN-1000, while blood chemistry was evaluated using the Roche Cobas c702. Before chemotherapy, all patients underwent staging screening using computed tomography of the abdomen and thorax to determine the extent of the tumor. Additional imaging techniques, including magnetic resonance imaging, bone scans, and positron emission tomography, were used based on the patient’s symptoms or as recommended by the attending physician. Baseline CT scans of the abdomen and thorax were carried out 1–3 weeks before starting chemotherapy, with follow-up imaging tests conducted every 8 ± 4 weeks after the commencement of chemotherapy. The Response Evaluation Criteria for Solid Tumors (RECIST) were used to assess radiological responses.

The inclusion criteria were as follows: (a) patients diagnosed with histopathological stage IV colorectal cancer, (b) patients receiving anti-EGFR therapy in combination with first-line chemotherapy, (c) patients whose GINI index can be calculated from the laboratory parameters analyzed at the time of diagnosis, and (d) patients with complete clinical records including demographics, pathology, and treatment modalities.

The exclusion criteria were as follows: (a) patients with clinically proven acute or chronic infection, systemic inflammation, or other autoimmune conditions; (b) patients receiving immunosuppressive therapy; (c) patients afflicted with hematological disorders; (d) patients with a confirmed second malignant neoplasm from different sites; (e) patients who had received a transfusion of a blood product in the month before cancer treatment; and (f) patients who used an enteral nutrition solution.

### Statistical methods

The Shapiro–Wilk test was used; histograms and q–q plots were examined to confirm the normality of the data. Differences between the groups were compared using Fisher’s exact test and Pearson’s χ2 test for categorical variables. Values are expressed as frequency and percentage, mean and standard deviation, or median and minimum–maximum. Receiver operating characteristic (ROC) curves were used to detect the discriminative impact of the GINI and NLR in predicting the survival rate of patients with metastatic cancer.

Progression-free survival (PFS) was calculated by recording the time from the start of first-line therapy to the date of progression in months. Metastatic overall survival (mOS) was calculated by recording the time from the start date of first-line therapy to the date of death or the last follow-up in months. The risk factors for PFS and mOS were identified using univariate and multivariate Cox regression analyses. The Kaplan-Meier method was applied to determine the probability of PFS and mOS, and the log-rank test was performed for group comparisons.

The hazard ratio (relative risk) was obtained by taking the 95% confidence interval, and a p-value of 0.05 was considered statistically significant. Variables significant at *p* < 0.05 level were included in a multivariate model, and backward stepwise selection was used at the *p* < 0.10 stringency level to determine the independent risk factors. ROC curves were calculated with 95% confidence intervals and compared using DeLong’s test. For each marker, cut-off values were determined using the Youden index. Using these cut-off values for each marker, sensitivity, specificity, and positive and negative predictive values were calculated with 95% confidence intervals. Analyses were conducted using R 4.3.3. (www.r-project.org), pROC^22^, MVN^23^, and easy ROC 1.2^24^ software. A *P-value* of less than 5% was considered statistically significant.

## Results

The study included 177 patients with metastatic colorectal adenocarcinoma (mCRC): 118 (66.7%) men and 59 (33.3%) women. The median age of the patients was 63 years (31–85 years). The tumors were located in the right colon in 30 patients (16%) and in the left colon in 147 patients (83.1%). The most common site of metastasis was the liver in 116 (65.5%) patients, followed by the lungs in 49 (27.7%) patients, and peritoneum in 24 (13.6%) patients. First-line chemotherapy; 97 patients received FOLFİRİ (Leucovorin + Irinotecan + Fluorouracil), 80 patients received oxaliplatin based (FOLFOX (Oxaliplatin + Leucovorin + Fluorouracil) or XELOX (Oxaliplatin + Capecitabine).

**Fig. 1:**
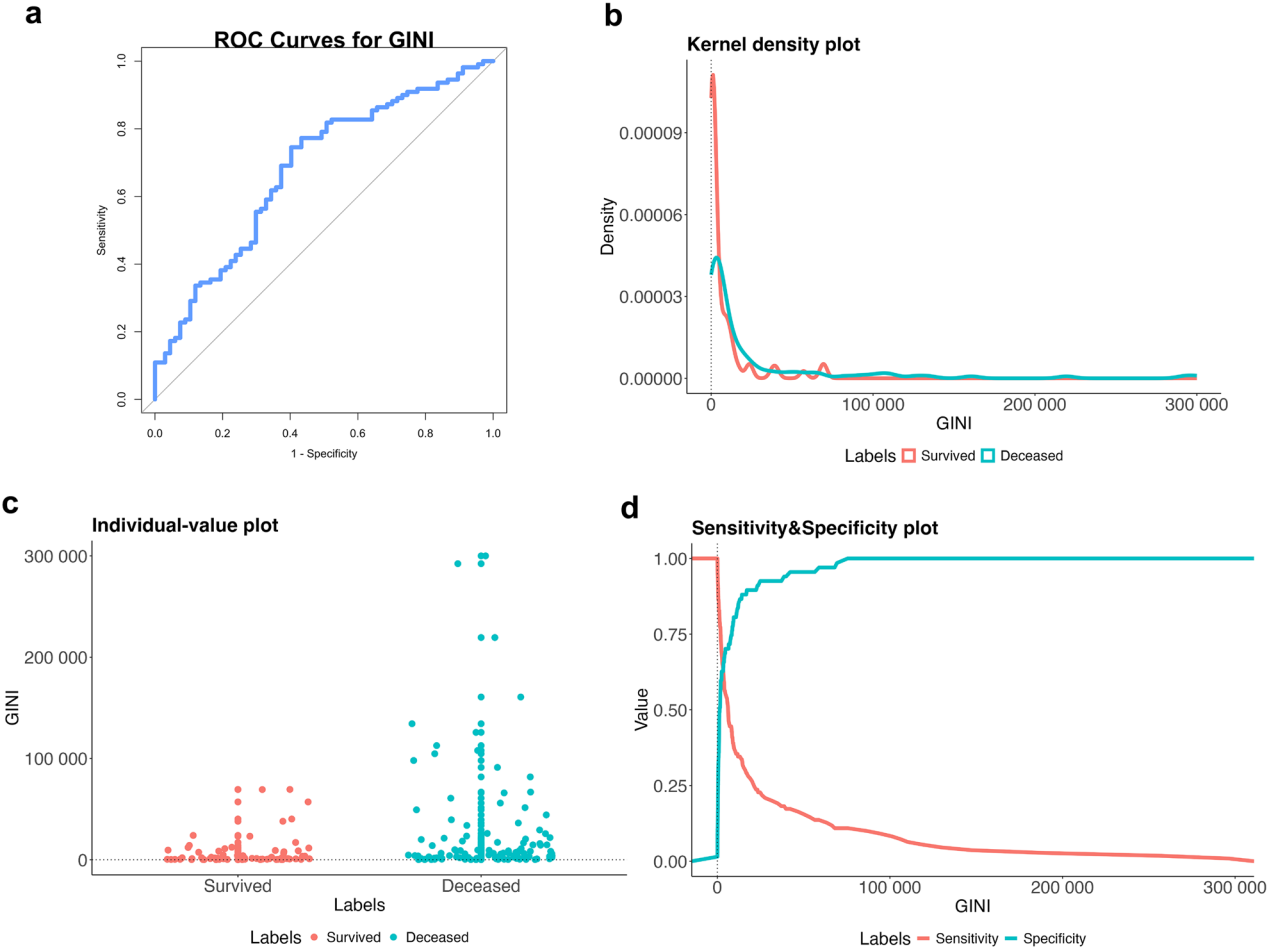
(**a**) Receiver operating characteristic curve analysis results depicting the association between the Global Immune-Nutrition-Inflammation Index values and survival outcomes. (**b**) In the kernel density plot of the GINI variable, the red line represents the Survived group and the blue line represents the Deceased group. GINI values are concentrated at lower levels in both groups, showing a right-skewed distribution. The Survived group has a sharper and higher peak, while the Deceased group shows a more spread-out distribution. (**c**) In the individual-value plot of the GINI variable, red dots represent the Survived group and blue dots represent the Deceased group. GINI values in the Survived group are mostly concentrated at lower levels and within a narrower range. In contrast, the Deceased group shows a wider spread, with many individuals having much higher GINI values. (**d**) In the sensitivity and specificity plot of the GINI variable, the red line represents sensitivity and the blue line represents specificity. Sensitivity is highest at low GINI values and decreases steadily as GINI increases. In contrast, specificity starts low and rises sharply, reaching near-perfect levels at higher GINI values.

In this study, 97 patients were identified as having wild-type BRAF, while BRAF results were unavailable for the other 80 patients. Based on the threshold values established through ROC curve analysis, the GINI and NLR were categorized into two groups: <2000 and ≥ 2000 (with a sensitivity of 74.5% and specificity of 59.7%) (Fig. 1(a, b,c, d), Table 4), and ≤ 2.71 and > 2.71 (with a sensitivity of 60% and specificity of 51%), respectively.

The baseline characteristics of the patients with mCRC treated with pre-chemotherapy GINI are shown in Table 1.

**Table 1:** Characteristics of participants according to GINI.

Variables	GINI Group		Total (*n* = 177)	*p*-value
	< 2000 (*n* = 69)	≥ 2000 (*n* = 108)		
**Gender** Female Male	29(42.0) 40(58.0)	30(27.8) 78(72.2)	59(33.3) 118(66.7)	**0.050**
**Age (years)** <65 ≥65	39(56.5) 30(43.5)	59(54.6) 49(45.4)	98(55.4) 79(44.6)	0.805
**ECOG performance status** 0–1 2	61(88.4) 8(11.6)	84(77.8) 24(22.2)	145(81.9) 32(18.1)	0.073
**Metastatic site** Liver Lung Peritoneum	44(63.8) 21(30.4) 5(7.2)	72(66.7) 28(25.9) 19(17.6)	116(65.5) 49(27.7) 24(13.6)	0.692 0.513 **0.050**
**Number of metastatic sites** Single site Multiple	16(23.2) 16(23.2)	16(14.8) 92(85.2)	32(18.1) 145(81.9)	0.158
**Chemotherapy** FOLFIRI^a^ Oxaliplatin based^b^	38(55.1) 31(44.9)	59(54.6) 49(45.4)	97(54.8) 80(45.2)	0.954
**Tumors site** Right colon Left colon	11(15.9) 58(84.1)	19(17.6) 89(82.4)	30(16.9) 147(83.1)	0.775
**Anti-EGFR therapy** Panitumumab Cetuximab	23(33.3) 46(66.7)	44(40.7) 64(59.3)	67(37.9) 110(62.1)	0.322
**Lactate dehydrogenase (LDH)** ^c^ < ULN ≥ULN	45(65.2) 24(34.8)	53(49.1) 55(50.9)	98(55.4) 79(44.6)	**0.035**
**Albumin** <4 g/d ≥4 g/d	67(97.1) 2(2.9)	82(75.9) 26(24.1)	149(84.2) 28(15.8)	**< 0.001**
**CEA (ng/ml)** ^**d**^ < ULN ≥ULN	38(55.1) 31(44.9)	48(44.4) 60(55.6)	86(48.6) 91(51.4)	0.168
**BMI(kg/m2)** <25 ≥25	25(36.2) 44(63.8)	50(46.3) 58(53.7)	75(42.4) 102(57.6)	0.186
**Adjuvant chemotherapy** Yes No	47(68.1) 22(31.9)	62(57.4) 46(42.6)	109(61.6) 68(38.4)	0.153
**Comorbidity** Yes No	45(65.2) 24(34.8)	73(67.6) 35(32.4)	118(66.7) 59(33.3)	0.744

Values are expressed as n(%). ^a^Folfiri(Irinotecan + Leucovorin + Fluorouracil), ^b^Xelox(Oxaliplatin + Capecitabine), ^b^Folfox(Oxaliplatin + Leucovorin + Fluorouracil) ^c^Upper limit of reference range: 250 U/L; ^d^Upper limit of reference range: 6.5 ng/ml ULN: Upper limit of normal; ECOG: Eastern Cooperative Oncology Group. Statistically significant *p* values are shown in bold.

Univariate analyses of PFS confirmed significant associations with performance status (*p* = 0.018), the number of metastatic sites (*p* = 0.035), albumin level (*p* < 0.001), and pre-chemotherapy GINI (*p* < 0.001). Furthermore, multivariate analysis revealed that albumin (HR 1.83, *p* = 0.009) and pre-chemotherapy GINI (HR 1.79, *p* = 0.004) served as significant independent prognostic indicators for PFS (Table 2).

**Table 2:** Univariate and multiple Cox regression analysis of variables for PFS.

Variables	Univariate		Multiple	
	HR (95%CI)	*p*-value	HR (95%CI)	*p*-value
**Age (years)** <65 ≥65	1.00 0.82(0.57–1.17)	- 0.264	-	-
**Gender** Female Male	1.00 0.77(0.53–1.10)	- 0.149	-	-
**ECOG performance status** 0–1 2	1.00 1.65(1.09–2.50)	- **0.018**	-	-
**Chemotherapy** FOLFIRI^a^ Oxaliplatin based^b^	1.00 1.04(0.73–1.48)	- 0.830	-	-
**Anti-EGFR therapy** Panitumumab Cetuximab	1.00 1.15(0.80–1.66)	- 0.441	-	-
**Number of metastatic sites** Single site Multiple	1.00 1.70(1.03–2.81)	- **0.038**	-	-
**Lactate dehydrogenase (LDH)** ^c^ < ULN ≥ULN	1.00 1.31(0.92–1.86)	- 0.138	-	-
**Albumin** <4 g/d ≥4 g/d	1.00 2.25(1.45–3.49)	- **< 0.001**	1.00 1.83(1.16–2.88)	- **< 0.009**
**Skin toxicity** Present Absent	1.00 1.39(0.98–1.98)	- 0.067	-	-
**Tumors site** Right colon Left colon	1.00 1.08(0.68–1.72)	- 0.735	-	-
**CEA (ng/ml)** ^**d**^ < ULN ≥ULN	1.00 1.38(0.97–1.96)	- 0.078	-	-
**BMI(kg/m2)** <25 ≥25	1.00 0.94(0.66–1.34)	- 0.713	-	-
**Comorbidity** No Yes	1.00 0.95(0.65–1.39)	- 0.795	-	-
**NLR** ≤2.71 >2.71	1.00 1.41(0.98–2.02)	- 0.063	-	-
**GINI** < 2000 ≥ 2000	1.00 2.01(1.37–2.95)	- **< 0.001**	1.00 1.79(1.20–2.67)	- **< 0.004**

CI: Confidence interval; HR: Hazard ratio; PFS: Progression-free survival; ECOG PS: Eastern Cooperative Oncology Group performance status; ^a^Folfiri(Irinotecan + Leucovorin + Fluorouracil), ^b^Xelox(Oxaliplatin + Capecitabine), ^b^Folfox(Oxaliplatin + Leucovorin + Fluorouracil) ^c^Upper limit of reference range: 250 U/L; ^d^Upper limit of reference range: 6.5 ng/ml; ULN: Upper limit of normal; NLR: neutrophil to lymphocyte ratio GINI: Global immune-nutrition-inflammation index. Statistically significant p values are shown in bold.

Univariate analysis of OS confirmed that performance status (*p* = 0.003), number of metastatic sites(*p* = 0.040), lactate dehydrogenase (*p* = 0.002), albumin (*p* < 0.001), CEA (*p* = 0.007), skin toxicity (*p* = 0.011), pre-chemotherapy NLR (*p* = 0.015), and pre-chemotherapy GINI (*p* < 0.001) were significantly associated with survival. Multivariate analysis for OS confirmed that performance status (HR, 1.65; *p* = 0.018), number of metastatic sites (HR, 1.74, 0.005), skin toxicity (HR, 1.79, *p* = 0.003), pre-chemotherapy NLR (HR, 1.49, *p* = 0.045), and pre-chemotherapy GINI (HR, 3.25, *p* < 0.001) were significant independent prognostic indicators (Table 3).

**Table 3:** Univariate and multiple Cox regression analysis of variables for mOS.

Variables	Univariate		Multiple	
	HR (95%CI)	*p*-value	HR (95%CI)	*p*-value
**Age (years)** <65 ≥65	1.00 0.73(0.49–1.07)	- 0.103	-	**-**
**Gender** Female Male	1.00 0.82(0.56–1.21)	- 0.321	-	**-**
**ECOG performance status** 0–1 2	1.00 1.88(1.24–2.84)	- **0.003**	1.00 1.65(1.09–2.50)	**0.018**
**Chemotherapy** FOLFIRI^a^ Oxaliplatin based^b^	1.00 1.08(0.74–1.58)	- 0.680	-	**-**
**Anti-EGFR therapy** Panitumumab Cetuximab	1.00 0.98(0.66–1.44)	- 0.909	-	**-**
**Number of metastatic sites** Single site Multiple	1.00 1.78(1.03–3.1)	- **0.040**	1.00 1.74(1.18–2.57)	**0.005**
**Lactate dehydrogenase (LDH)** ^c^ < ULN ≥ULN	1.00 1.82(1.24–2.67)	- **0.002**		
**Albumin** <4 g/d ≥4 g/d	1.00 2.62(1.66–4.12)	- **< 0.001**	-	**-**
**Skin toxicity** Present Absent	1.00 1.64(1.12–2.41)	- **0.011**	1.00 1.79(1.22–2.62)	**0.003**
**Tumors site** Right colon Left colon	1.00 0.93(0.56–1.54)	- 0.778	-	**-**
**CEA (ng/ml)** ^**d**^ < ULN ≥ULN	1.00 1.69(1.15–2.47)	- **0.007**		
**BMI(kg/m2)** <25 ≥25	1.00 0.71(0.49–1.04)	- 0.081	-	**-**
**Comorbidity** No Yes	1.00 0.87(0.58–1.3)	- 0.493	-	**-**
**NLR** ≤2.71 >2.71	1.00 1.62(1.1–2.38)	- **0.015**	1.00 1.49(1.01–2.22)	**0.045**
**GINI** < 2000 ≥ 2000	1.00 3.38(2.12–5.39)	- **< 0.001**	1.00 3.25(2.00-5.27)	**< 0.001**

CI: Confidence interval; HR: Hazard ratio; mOS: Metastatic overall survival; ECOG PS: Eastern Cooperative Oncology Group Performance Status; ^a^Folfiri(Irinotecan + Leucovorin + Fluorouracil), ^b^Xelox(Oxaliplatin + Capecitabine), ^b^Folfox(Oxaliplatin + Leucovorin + Fluorouracil) ^c^Upper limit of reference range: 250 U/L; ^d^Upper limit of reference range: 6.5 ng/ml; ULN: Upper limit of normal; NLR: neutrophil to lymphocyte ratio GINI: Global immune-nutrition-inflammation index. Statistically significant p values are shown in bold.

**Table 4:** ROC analysis results.

	ROC statistic		Diagnostic Statistics			
	AUC(%95 CI)	*p*	Sensitivity	Specificity	LR+	LR-
**GINI > = 2000**	0.68(0.60–0.76)	**< 0.001**	74.5(65.4–82.4)	59.7(47.0-71.5)	1.85	0.43

**Fig. 2:**
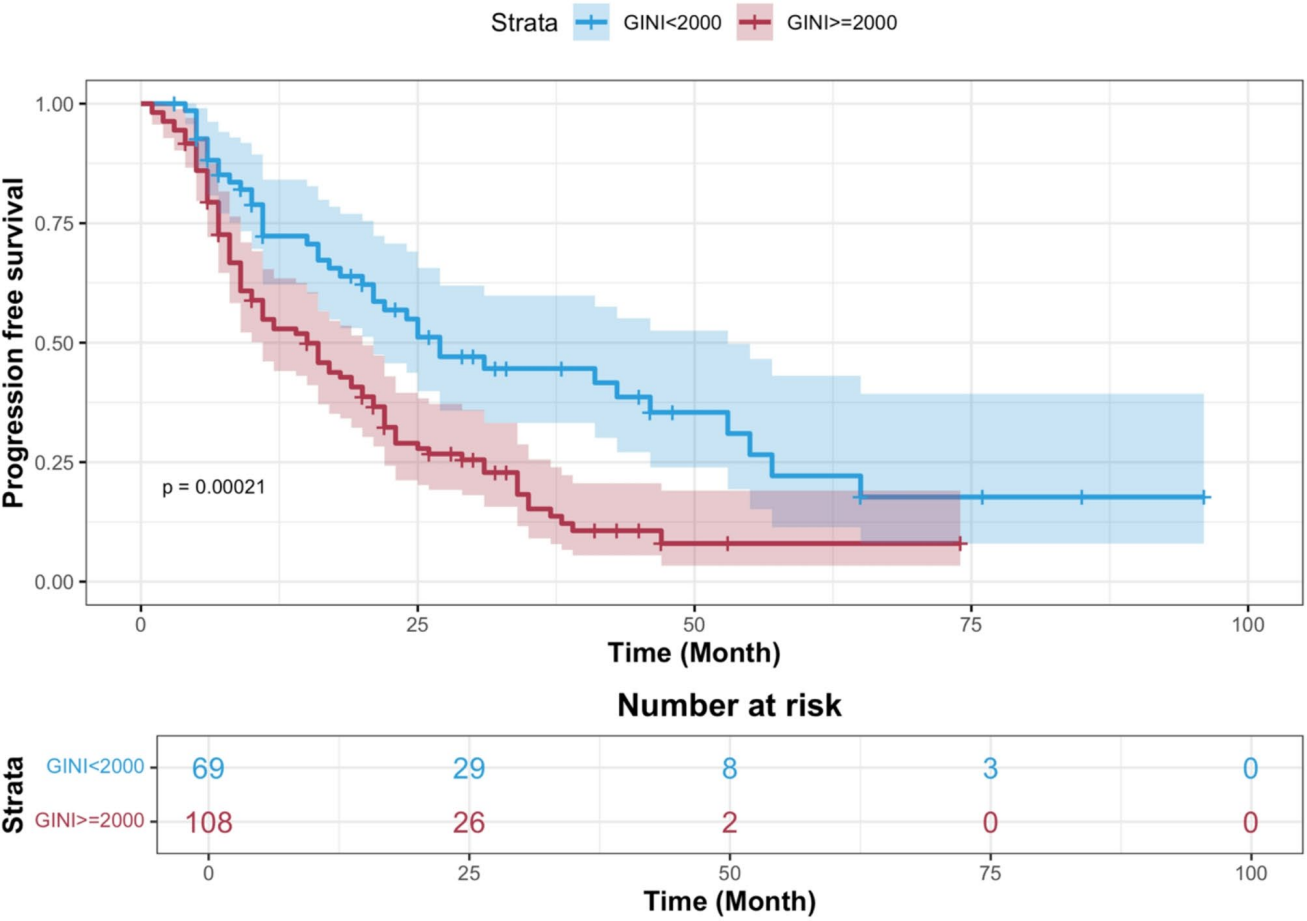
Kaplan-Meier curves of PFS according to global ımmune-nutrition-ınflammation Index groups.

**Fig. 3:**
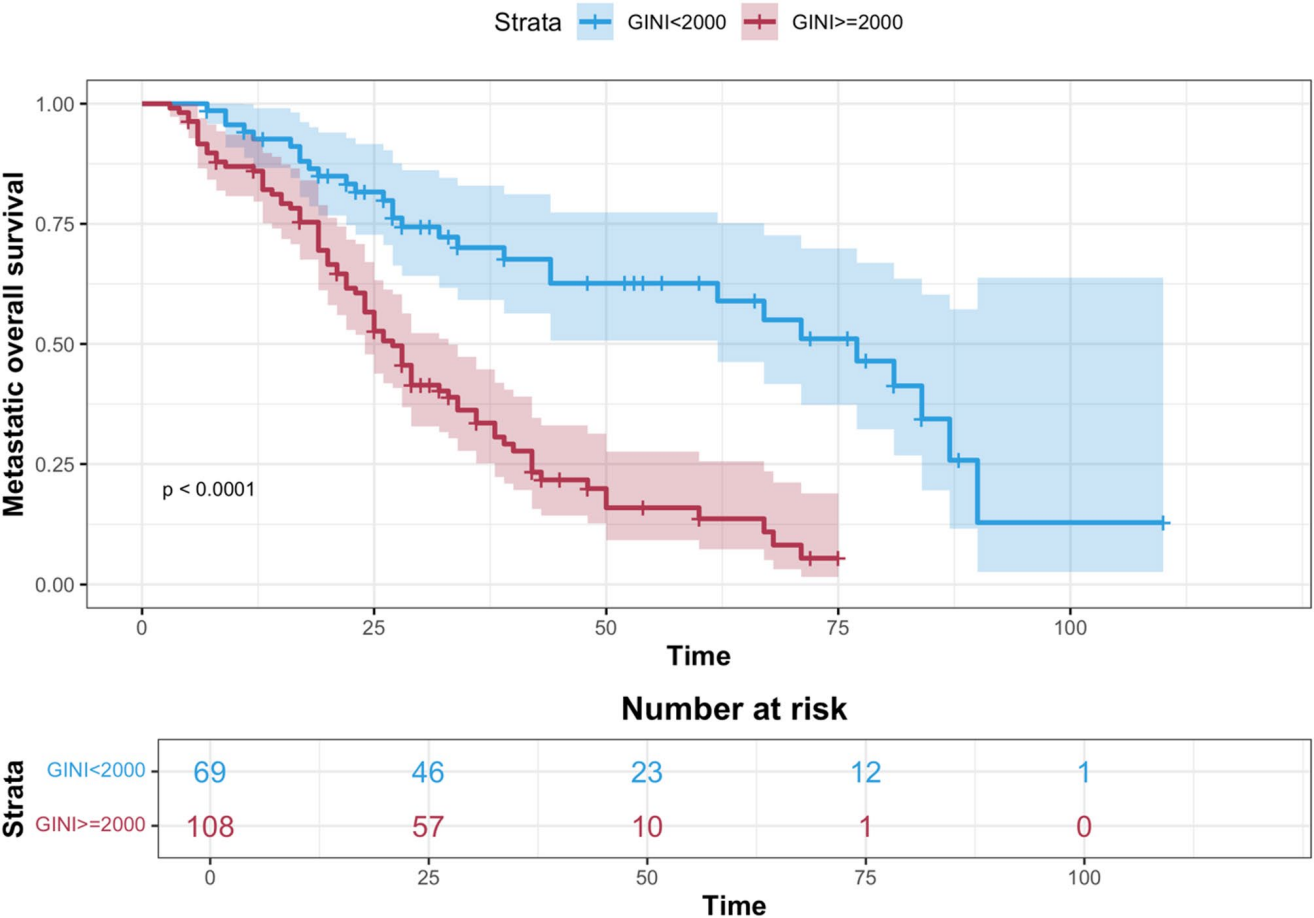
Kaplan–Meier curves of mOS according to Global Immune-Nutrition-Inflammation Index groups.

The median PFS was 15 months (95% confidence interval (CI): 10.5–19.48 in the group with GINI ≥ 2000 and 27 months (95% CI: 17.5–36.4) in the group with GINI < 2000 (*p* = 0.00021) (Fig. 2). The median OS was 27 months (95% CI: 23.8–30.1) in the group with GINI ≥ 2000 and 77 months (95% CI: 59.7–94.2) in the group with GINI < 2000 (*p* < 0.001) Fig. 3).

## Discussion

There is growing evidence indicating that persistent inflammation is crucial in the initiation and progression of cancers^25^. Inflammation also has an impact on the immune microenvironment of the tumor and the response to therapy.

Malnutrition is common among cancer patients. Malnutrition adversely affects both the effectiveness of treatment and the patient’s overall quality of life. This condition may arise due to the tumor itself, the patient’s reaction to the tumor, or the treatments aimed at combating cancer^26^.

Unfortunately, malnutrition is greatly underestimated in most oncology units and malnutrition associated with colorectal cancer can have serious consequences if left untreated, leading to reduced patient tolerance to chemotherapy cycles and adverse effects on patient outcomes^27,28^.

Recent research has shown that immune and nutritional status as well as clinicopathological parameters are important predictors of tumor outcome. Immunonutritional indices, which assess inflammatory markers and nutritional status together, are prognostic factors in many cancers. The prognostic importance of PNI, an immune nutritional index, has been demonstrated in patients with metastatic colorectal cancer^19^. The CONUT score is another immune nutritional index used to assess blood components, such as lymphocyte number, albumin, and total cholesterol^20^. The prognostic significance of CONUT score in gastrointestinal cancer has been described in several reports^29^.

Topkan et al. recently highlighted the prognostic significance of the GINI, an immune nutritional index, in individuals with stage 3 non-small cell lung cancer undergoing chemoradiotherapy^21^. Patients were divided into low and high GINI categories based on a cut-off value of 1562, established through ROC curve analysis. They observed notable differences in median OS and 5-year OS between these two groups.

A different study indicated that the GINI serves as a useful prognostic marker for individuals with non-metastatic stomach cancer. Patients were categorized into low and high GINI groups using a threshold of 1.730. Significant variations in overall survival (OS) rates were observed between these groups. Yamamoto et al. demonstrated that GINI was an independent prognostic factor for OS in esophageal cancer patients undergoing curative treatment^30^. In our research, based on thresholds established through ROC curve analysis, GINI was split into two categories: GINI < 2000 and GINI ≥ 2000. The present investigation revealed that an elevated GINI value correlated with diminished progression-free survival (PFS) (*p* = 0.001) and overall survival (OS) (*p* < 0.001) in initial univariate assessments. Subsequent multivariate analysis further established high GINI as a significant independent prognostic factor for OS (HR: 3.25, 95% CI: 2.00-5.27, *p* < 0.001) and PFS (HR: 1.79, 95% CI: 1.20–2.67, *p* = 0.004).

Recently, NLR has been proposed as a significant marker of inflammation and a possible indicator of long-term outcomes in patients with CRC^31^. In this study, patients with an NLR greater than 2.71 exhibited notably worse overall survival (OS) outcomes (*p* = 0.015) in univariate analyses. Furthermore, NLR was identified as an independent predictor of survival in multivariate analysis (*p* = 0.045; HR: 1.49; 95% CI: 1.01–2.22).

Skin toxicity (ST) is the most prevalent side effect associated with EGFR inhibitors. The exact mechanisms causing skin-related toxicity from anti-EGFR treatment remain partially unclear. EGFR is highly expressed in skin follicles, keratinocytes and hair follicles, and inhibiting EGFR signaling in these cells results in apoptosis^32,33^. The success of anti-EGFR therapy in patients with mCRC has been correlated with the severity of ST^33–35^. A phase 3, controlled, randomized, multicenter study demonstrated that the combination of panitumumab with best supportive care resulted in better PFS compared to BSC alone in patients with chemorefractory metastatic colorectal cancer (mCRC). Exploratory analyses revealed that patients experiencing more severe ST had a significantly longer OS^36^.

The study indicated that skin toxicity was linked to worse overall survival (OS) in the initial analysis (*p* = 0.011). Importantly, skin toxicity was also identified as an independent predictor of OS in the multivariate analysis, with a hazard ratio (HR) of 1.79 and a 95% confidence interval (CI) of 1.22–2.62 (*p* = 0.003).

The ECOG-PS is closely linked to the prognosis of different cancer tissue types, including CRC^37,38^. In this study, Cox multivariate survival analysis revealed that poor PS was an independent adverse prognostic factor for OS (HR, 1.65; 95% CI: 1.09–2.50, *p* = 0.018) in mCRC patients.

The number of metastatic sites is a recognized prognostic indicator for individuals with advanced colorectal cancer^39^. This study found that an increased number of metastatic sites was associated with worse PFS and OS in the univariate analysis. In addition, the number of metastatic sites was identified as an independent predictor of OS (HR, 1.74; 95% CI: 1.18–2.57, *p* = 0.005).

This study has certain limitations. The design introduces the potential for selection bias and uncontrolled confounding factors, meaning the results may reflect specific institutional treatment patterns rather than broader trends. Furthermore, although the GINI index has been investigated for use in predicting the prognosis of various cancers, there is currently no consensus on the exact cut-off level for different cancer types^21,30,40^. The use of ROC-derived cut-off values in this study lacks external validation, which could limit the reproducibility of our findings. Therefore, before this marker can be used in routine clinical practice, we strongly recommend that these results be validated prospectively in larger, multicenter clinical trials, in order to fully assess the clinical utility of the GINI index in patients with mCRC. Additionally, we acknowledge that our analysis did not consider all the variables that could influence GINI index results.

In conclusion, the present data showed that albumin level and pre-chemotherapy GINI were independent prognostic tools for PFS, and performance status, number of metastatic sites, skin toxicity, pre-chemotherapy NLR, and pre-chemotherapy GINI were substantial independent prognostic tools for OS. We believe that GINI could play a crucial role in predicting patient outcomes because it encompasses both inflammatory and nutritional factors. However, larger prospective studies should be conducted to confirm whether the GINI has prognostic and predictive properties in patients with mCRC.

## Data Availability

The data that support the findings of this study are available from the corresponding author upon reasonable request.
